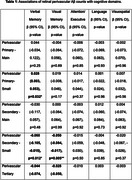# Linking retinal amyloid imaging to cognition and hippocampal integrity – a retrospective cohort analysis

**DOI:** 10.1002/alz70856_102028

**Published:** 2025-12-25

**Authors:** Mitzi M. Gonzales, Dieu‐Trang Fuchs, Jonah Doustar, Yosef Koronyo, Dale S. Sherman, Patrick Lyden, Nancy Sicotte, Keith L Black, Sarah Kremen, Oana M Dumitrascu, Maya Koronyo‐Hamaoui

**Affiliations:** ^1^ Cedars‐Sinai Medical Center, Los Angeles, CA, USA; ^2^ Department of Neurosurgery, Maxine Dunitz Neurosurgical Research Institute, Cedars‐Sinai Medical Center, Los Angeles, CA, USA; ^3^ University of Southern California, Los Angeles, CA, USA; ^4^ Mayo Clinic College of Medicine and Science, Scottsdale, AZ, USA; ^5^ Department of Neurology, Cedars‐Sinai Medical Center, Los Angeles, CA, USA

## Abstract

**Background:**

Sensitive, accessible biomarkers of the pathologies underlying Alzheimer's disease and related dementias (ADRD) are critical for accurate diagnosis, deeper understanding of disease mechanisms, and development of new therapies with validated target engagement. The retina is connected to the brain via the optic nerve, which may facilitate the transmission and spread of amyloid beta (Aβ) and tau species. In postmortem studies, retinal Aβ deposits have been associated with neuritic Aβ‐plaque and neurofibrillary tangle burden, particularly in the entorhinal and temporal cortices. The goal of the current study was to evaluate associations between retinal perivascular amyloid and cognition, as well as to explore effect modification by hippocampal volumetry.

**Method:**

Participants, aged ≥40 years, underwent retinal imaging, brain MRI, FDG‐PET, and a comprehensive neuropsychological assessment. Retinal amyloid imaging was conducted following curcumin ingestion and pupil dilation using a confocal scanning laser ophthalmoscope. Perivascular Aβ plaque counts were quantified in the superotemporal peripheral region following manual identification and tracking of retinal arteries and veins. Associations between retinal perivascular Aβ plaque counts with cognitive domains were evaluated using linear regression models adjusted for age, sex, and estimated premorbid functioning. Where significant associations were detected (*p*‐value<0.05), effect modification by hippocampal volumetry was examined with additional adjustment for intracranial volume.

**Result:**

The sample included 26 participants (mean age 65±7 years, 50% female). Higher amyloid plaque counts in the perivascular secondary small and tertiary branches and in the periarteriolar tertiary branch were associated with poorer verbal and visual memory (Table 1). Significant interactions were observed between amyloid plaque count in the perivascular secondary small branch (β(95% Confidence Interval) = 0.397(0.037‐0.756), *p* = 0.033) and periarteriolar tertiary branch (β(95% Confidence Interval) = 0.756(0.165–1.346), *p* = 0.017) with hippocampal volumetry for visual memory.

**Conclusion:**

Our pilot study provides preliminary evidence that perivascular Aβ accumulation in smaller vessels of the superotemporal retina may be a marker of memory impairment that may interact synergistically with hippocampal atrophy. Longitudinal studies consisting of multimodal ophthalmic assessments, brain imaging, ADRD biofluid markers, and cognitive testing will be necessary to capture the temporal sequence of retinal pathological changes and their associations with those in the brain.